# Genome-wide analysis of chromatin features identifies histone modification sensitive and insensitive yeast transcription factors

**DOI:** 10.1186/gb-2011-12-11-r111

**Published:** 2011-11-07

**Authors:** Chao Cheng, Chong Shou, Kevin Y Yip, Mark B Gerstein

**Affiliations:** 1 Department of Molecular Biophysics and Biochemistry, Yale University, 260 Whitney Avenue, New Haven, CT 06520, USA; 2 Program in Computational Biology and Bioinformatics, Yale University, 260 Whitney Avenue, New Haven, CT 06520, USA; 3 Department of Computer Science and Engineering, The Chinese University of Hong Kong, Rm 1006, Ho Sin-Hang Engineering Bldg, Shatin, New Territories, Hong Kong; 4 Department of Computer Science, Yale University, 260 Whitney Avenue, New Haven, CT 06520, USA

## Abstract

We propose a method to predict yeast transcription factor targets by integrating histone modification profiles with transcription factor binding motif information. It shows improved predictive power compared to a binding motif-only method. We find that transcription factors cluster into histone-sensitive and -insensitive classes. The target genes of histone-sensitive transcription factors have stronger histone modification signals than those of histone-insensitive ones. The two classes also differ in tendency to interact with histone modifiers, degree of connectivity in protein-protein interaction networks, position in the transcriptional regulation hierarchy, and in a number of additional features, indicating possible differences in their transcriptional regulation mechanisms.

## Background

Transcription factors regulate target gene expression through binding to specific genomic regions. In *Saccharomyces cerevisiae*, transcription factor (TF) binding sites (TFBSs) are often adjacent to and upstream of target loci due to the compact nature of the yeast genome [1,2]. Upon binding, TFs interact with RNA polymerase II to activate or repress transcription. TFs also recruit chromatin modification enzymes to induce chromatin structure changes, which in turn affect the accessibility of factors to genomic DNA regions [3,4]. The target genes of a TF change according to developmental, physiological and extracellular environmental conditions [5]. In addition, TFs interact with each other through combinatorial binding [6]. Uncovering TF target genes and inter-relationships between TFs for all different conditions is thus important for understanding gene expression regulation, but it is also a difficult task due to the scale of the problem.

Several different experimental methods have been developed to identify TFBSs. Chromatin immunoprecipitation followed by tiling array (ChIP-chip) has been widely used to identify TFBSs at the genomic scale [7-9]. More recently, high-throughput sequencing after chromatin immunoprecipitation (ChIP-Seq) has shown promise in identifying TFBSs at higher resolutions [10,11]. With these methods, increasing amounts of TFBS data have been accumulated for different TFs in different species, cell types, conditions, and so on, which have started to unravel the global picture of gene expression regulation. In yeast, the TFBSs and target genes for an almost complete set of TFs have been mapped in common YPD medium using ChIP-chip [5]. However, resources are still too limited to support a complete exploration of TF binding for all the combinations of cell types and conditions.

Many computational methods have also been proposed to predict TFBSs [12-19]. These methods are mostly based on the idea that the binding of a TF is mediated by the recognition of its binding motif represented as a position-specific scoring matrix (PSSM). PSSMs are usually discovered as enriched motifs from TFBSs in ChIP-chip or ChIP-seq experiments, or *de novo *from non-coding genomic sequences [5,20]. Scanning and matching PSSMs in the genome constitute the core of these methods, which are then improved by incorporating information on motif conservation and TFBS co-localization. Nevertheless, these methods often lead to considerably high rates of false positives. Furthermore, most of these methods are not condition specific and thus do not reflect the dynamic nature of TF binding under different conditions.

Chromatin modifications can modulate the accessibility of DNA regions and affect the recruitment of TFs [3,4,21]. Both functions directly relate to transcription regulation by TFs. Genomic mapping of chromatin modifications in yeast using ChIP-chip has provided the opportunity to investigate their underlying relationships with TFBSs [22,23]. Many chromatin modifications have been shown to be associated with transcription activation and repression [3,4]. Recent studies have shown that incorporating histone modification data improves prediction of TFBSs in mouse and human [24,25]. In these models, chromatin modifications generally provide non-TF-specific chromatin accessibility, while PSSMs determine TF-specific bindings.

Here we propose a method that integrates PSSMs and chromatin modifications to improve TF target gene predictions in yeast. Specifically, we trained individual support vector machine (SVM) models [26] for 203 yeast TFs using 2 types of features: the existence of PSSMs upstream of genes and chromatin modifications adjacent to the ATG start codons. The models were trained and tested using TF target genes from ChIP-chip experiments. Interestingly, we found that some yeast TFs were more sensitive to histone modifications than others: their target genes had relatively higher histone modification signals, and were better predicted when these signals were included in the models. We termed these two TF classes as histone-sensitive and histone-insensitive TFs for simplicity. We show that histone-sensitive and histone-insensitive TFs differ in many biological characteristics found in large-scale and small-scale experiments. Furthermore, we used the model to investigate condition specificity and TF-specificity of chromatin modifications as well as TF-TF co-operation. Our analysis helps understand the mechanism of gene expression regulation by TFs and chromatin modifications.

## Results

### Differential histone modifications between ChIP-chip verified and non-verified TFBSs

**Figure 1:**
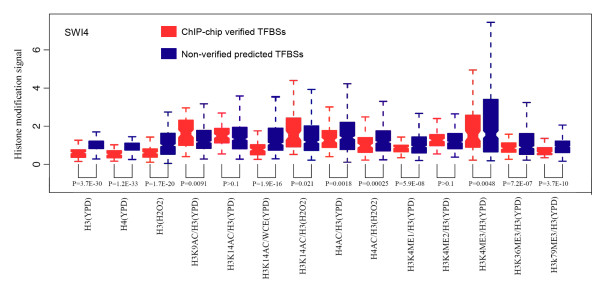
**Differential histone occupation and modifications between ChIP-chip verified TFBSs and non-verified motif matching sites**. Showing SWI4 as an example, most histone modifications (in different colors) are significantly different between ChIP-chip verified TFBSs (left boxes), which have binding motifs and are bound by TFs in ChIP-chip experiments, and non-verified motif matching sites (right boxes), which have matching motifs but are not bound by TFs.

In order to examine whether chromatin modifications are predictive features for functional TFBSs, we first investigated chromatin modification signals at ChIP-chip verified and non-verified TFBSs defined as follows. Based on previous TFBS prediction models, we denoted the TFBSs of a factor as the local genomic sequences that match its PSSM. We then used ChIP-chip experimental data to distinguish ChIP-chip verified and non-verified TFBSs based on the existence of actual binding peak signals. Although both verified and non-verified TFBSs contain TF binding PSSMs, they were found to have differential chromatin modification signals. Here, we use the factor SWI4, a component of the SBF complex regulating cell cycle gene expressions, as an example (Figure 1). We observed that individual histone modifications varied significantly between ChIP-chip verified and non-verified TFBSs. Among the 14 different histone modifications under 2 conditions (YPD and H_2_O_2_), 11 were significantly different (*P*-value < 0.01) in their signals between the verified and non-verified TFBSs of SWI4 (Figure 1). Among them, H3 and H4 signatures were particularly strong features for distinguishing between the two types of TFBSs, as they showed significantly lower signal in verified TFBSs than in non-verified TFBSs (*P*-value < 10^-20^). Consistent with previous studies, this indicates that verified binding sites of factors in regulatory regions are typically depleted of nucleosomes [27-30]. Encouraged by the observed differential histone modifications between verified and non-verified TFBSs, we then constructed a model that combines histone features with binding motif information for target gene prediction in yeast.

### Improving target gene prediction by combining histone modifications and PSSMs

**Figure 2:**
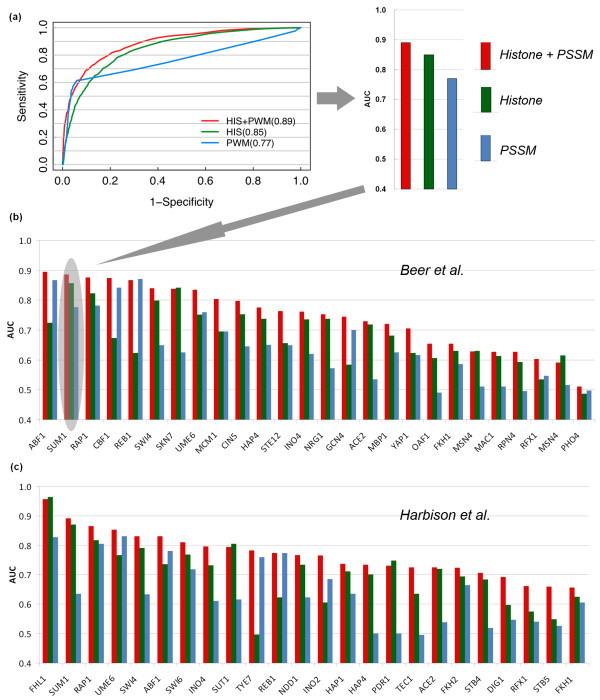
**Chromatin modifications substantially improve TF target gene predictions**. **(a) **ROC curves show improved TF target gene predictions using histone modifications. **(b, c) **Performance of prediction models for individual TFs, with PSSMs from Beer and Tavazoie [20] (b) and from Harbison *et al*. [5] (c). TFs are sorted by prediction performance using histone modifications and PSSMs (red bars).

Since the *S. cerevisiae *genome is quite compact with respect to other higher eukaryotic species, it is reasonable to define the target genes of a TF as those with one or more upstream TFBSs. We combined chromatin modifications and PSSM data and used them as input features to a SVM learning model for predicting TF target genes. The model was compared with two other models that are based only on histone modifications or PSSMs. The prediction accuracy of the models was tested using a gold standard data set from ChIP-chip experiments, which provided target genes of 203 yeast TFs [5]. Specifically, we choose 0.01 as the *P*-value cutoff for target gene calling from ChIP-chip, which provides us with enough high confidence positive target genes for model training (Figure 2a). The data set was separated into training and testing data, and the performance of the models was assessed by cross-validation (see Materials and methods).

For chromatin modifications, we used 11 histone modifications that covered most yeast ORF regions from ChIP-chip experiments [23]. Since TFs bind to the upstream sequence of ORFs, we focused on histone modification signals in the 1 kb of sequence flanking transcription start sites (ATGs), because TFBSs were enriched in these regions. For TF PSSMs, two independent sets were obtained from previous studies. One set comprised PSSMs discovered using a sequence analysis-based method, basically looking for enriched motifs in the DNA regions upstream of all yeast ORFs [20]. From approximately 5, 600 upstream sequences a total of 666 motifs have been discovered, among which 48 could be associated with known TFs according to the literature. The other set of PSSMs was based on ChIP-chip data [5]. For each TF a target gene set was determined and then the binding motif for the TF was identified from the DNA region upstream of these genes.

Our results indicate that, for almost all TFs, the model using both histone modification and PSSM data achieves the best or nearly best performance (measured using the area under the receiver operator characteristic (ROC) curve (AUC)) compared to the other two models using histone modification data or PSSM data alone (Figure 2; Additional files 1 and 2). For example, we obtained an AUC of 0.89 for predicting target genes of the factor SUM1 when both the histone modification and PSSM data were used. If only the PSSM or histone modification information was used, however, the models resulted in lower AUCs (0.77 for PSSM only and 0.85 for histone modification only; Figure 2a; Additional file 1). The improved performance of the combined model was observed for both of the TF PSSM sets (Figure 2b, c; Additional files 1 and 2), indicating that the improvement does not rely on a particular source or quality of PSSMs. Interestingly, for some TFs, the histone-only model performed better than the PSSM-only model, while for some other TFs, the opposite was observed.

**Figure 3:**
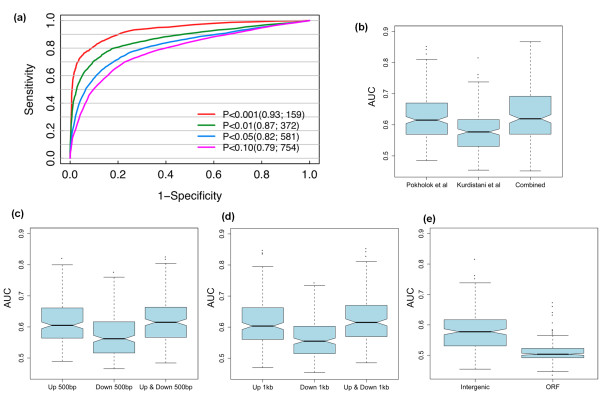
**Model parameters**. **(a) **Stricter thresholds for target gene calling result in better predictions. **(b) **Combination of independent histone modification data sets can improve target predictions. Predictions for 203 TFs are evaluated by area under the receiver operator characteristic (ROC) curve (AUC) for data from Pokholok *et al*. [23] and Kurdistani *et al*. [22]. **(c, d) **Using histone modifications within a 500-bp (c) and 1, 000-bp (d) window upstream and downstream of ATG sites of target genes achieves a similar performance to using only upstream signals, and better performance to using only downstream signals. There was no significant performance difference between using 500-bp and 1, 000-bp window sizes. **(e) **Using histone modifications in intergenic regions has better predictive power than using those in ORF coding regions.

In order to examine whether we could achieve better TF target prediction when we have more chromatin modification data, we used histone modification data sets from two independent experiments, performed by Pokholok *et al*. [23] and Kurdistani *et al*. respectively [22]. We found that we achieved a higher prediction accuracy by using the data set from Pokholok *et al*. than using that from Kurdistani *et al*. This is probably due to the fact that the latter contains only histone acetylation data, while the former contains both histone methylation and acetylation data, which might provide complementary information on the regulation of chromatin structure and TF binding. We then combined the two data sets for predicting TF target genes, and found that, for most TFs, the performance was only slightly better than using the Pokholok *et al*. data set alone (Figure 3b). Nevertheless, for some TFs we observed a substantial improvement when including the Kurdistani *et al*. data set (Table 1). It is thus promising that we might improve the performance of our chromatin model in the future by incorporating more histone modification data.

**Table 1:** Transcription factors with improved prediction by including multiple histone modification datasets

TF	AUC^a^	AUC^b^
CST6	0.70	0.80
IFH1	0.53	0.64
KSS1	0.53	0.65
RDS1	0.50	0.67
SFP1	0.59	0.74
SWI5	0.62	0.74
TYE7	0.53	0.64
YKL222C	0.57	0.69
YKR064W	0.49	0.59

^a^Data from Pokholok *et al*. [23]; ^b^data from Pokholok *et al*. [23] and Kurdistani *et al*. [22].

We also investigated the positional effect of histone modification signals for target gene prediction. First, we observed that signals of different types of histone modifications showed different patterns at DNA regions around the ATG, suggesting that they might affect TF binding in different ways. Second, histone modification signals upstream of the ATG are generally more predictive than those downstream of it, as we have observed for both 500-bp and 1, 000-bp flanking region sizes (Figure 3c, d). This is somewhat expected because TFBSs are more enriched in the upstream regions of ORFs for the purpose of transcriptional regulation. It is also interesting to see that ATG flanking regions of 500 bp and 1, 000 bp result in almost the same performance (Figure 3c, d). Given the compact nature of the yeast genome, transcription start sites for most ORFs are located within the region 1, 000 bp upstream of the ATG [31]. The comparable performance when using 500-bp ATG flanking region indicates that most discriminative histone modification signals for TF binding are embedded in this region. We also find that histone modifications in intergenic regions are more predictive than those in ORF coding regions (Figure 3e).

The number of PSSM-containing genes is another factor that might also affect prediction using histone modifications. We counted the number of genes with PSSMs in their promoters for each TF, and found no obvious relationship between this and the prediction power (AUC) when using histone modifications (Additional files 3 and 4). In fact, the number of PSSM-containing genes varies extensively from 79 to 5, 810 depending on the information content of the PSSMs, but its correlation with the prediction power is negligible (R = 0.1 for the Frankel *et al*. data set [32]).

Both histone modifications and TF binding are involved in regulation of gene expression. To rule out the possibility that the capability of histone modifications for predicting TF target genes is actually mediated by the expression level of genes, we examined the marginal effect of gene expression level on TF target prediction. Including expression levels as an additional predictor in the histone modification-based models does not significantly change the prediction accuracy for any TF. Furthermore, SVM models based on expression level alone can hardly predict targets for any of the TFs (Additional file 5). The only exception is FHL1 (prediction accuracy AUC = 0.79), which predominantly regulates the transcription of ribosomal protein genes; it is the extreme abundance of these genes that enables accurate prediction for this TF. Thus, the prediction power of histone modification models is not likely to be mediated by gene expression levels overall.

### Condition specificity of the chromatin model

TFs bind to and regulate target gene expression in a complex and dynamic manner to coordinate biological processes [5,33]. Chromatin modifications also change rapidly in response to stimuli from the extracellular environment [3]. Therefore, chromatin modifications in one condition should match TF target binding in that condition but not other conditions.

We investigated the condition specificity of our chromatin model in two conditions, YPD medium and H_2_O_2_. We tested a total of 12 TFs, for which we had the PSSM, histone modification and TF target binding data under both conditions. For each of the TFs, we constructed two separate chromatin models: one model (model A) used PSSM and histone modifications under the YPD condition as features, while the other (model B) used PSSMs from the YPD condition but histone modifications under the H_2_O_2 _condition. The two models were then used to predict TF target binding under H_2_O_2 _conditions. It is generally believed that TFs keep their binding specificity PSSMs in different conditions and even over large evolutionary distances. Therefore, we used PSSMs from the YPD condition as a close approximation in model B, where no PSSM information is available under the H_2_O_2 _condition.

**Figure 4:**
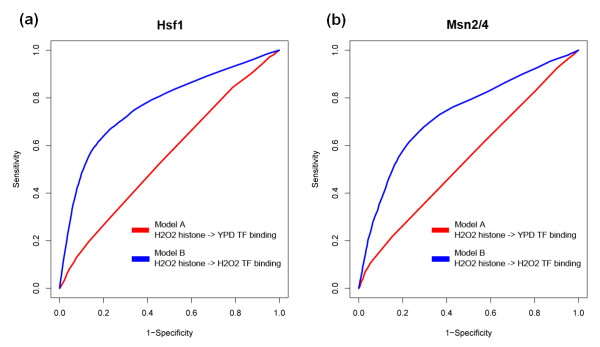
**Condition specificity of the chromatin model for TF target prediction**. **(a, b) **ROC curves showing the performance of two chromatin models in predicting target genes of HSF1 (a) and the MSN2/4 complex (b). The model (model B) using histone modifications in the H_2_O_2 _condition is better at predicting target genes in the H_2_O_2 _condition (blue curve) than in YPD medium (model A; red curve), which indicates condition specificity of chromatin modifications and TF target genes.

For TFs that are known to be functional under the H_2_O_2 _condition, model B performed better than model A. For example, HSF1, a heat shock TF that activates genes in response to stresses, is more active under the H_2_O_2 _condition, with 326 target genes, than in YPD medium, with only 123 target genes. Using condition-matched histone modification data (model B), our chromatin model achieved an AUC of 0.77. In contrast, using non-condition-matched data (model A), the chromatin model only achieved an AUC of 0.56 (Figure 4a). Similar results were observed for another TF, MSN2, which is activated along with MSN4 to regulate stress response genes. MSN2 is more active under the H_2_O_2 _condition, and our chromatin model performed better with condition-matched data (Figure 4b). These results indicate that histone modifications are actually dynamic and function in a condition-specific manner. Thus, target genes of TFs under a certain condition can be best predicted by using histone modification data from the same condition. In practice, this enables us to predict condition-specific target genes of TFs, which cannot be achieved using the PSSM-based method.

### Relative importance of different histone modifications for target prediction

TFs bind to the upstream regions of target genes by recognizing their specific binding motif PSSMs. We then asked an analogous question: do the binding sites of TFs have specific histone modification profiles? To address this question, we calculated a histone modification profile for each TF by averaging upstream ATG histone modification signals over all its target genes. In our analysis, we included 25 different histone modifications from the two studies mentioned above [22,23].

**Figure 5:**
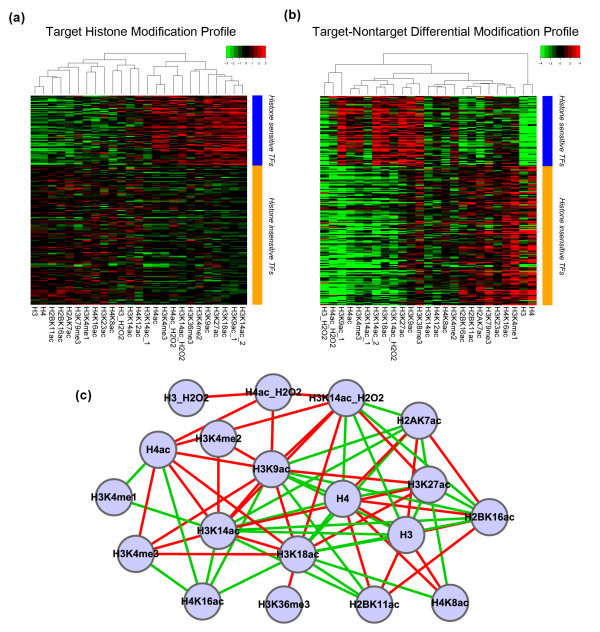
**Target histone modification profiles and target-non-target differential modification profiles of TFs**. **(a) **Target histone modification profiles comprising different normalized histone modification signals (columns) of TFs (rows). A target histone modification profile is the averaged histone modification signals of the TF's targets. TFs are clustered into histone-sensitive (blue bar) and -insensitive TFs (orange bar) using their target histone modification profiles. Histone-sensitive TFs have stronger histone modification signals. **(b) **Target-non-target differential modification profiles of TFs showing the discriminating power (t-statistic) of histone modifications to TF targets and non-targets. TFs are ordered the same as in (a). Histone-sensitive and -insensitive TFs have distinct differential modification profiles, indicating preferential histone modifications of TFs targets. **(c) **Correlation network of histone modifications in terms of TF differential modification profiles. Histone modification pairs with a correlation coefficient larger than 0.5 (red edges) or smaller than -0.5 (green edges) are connected. The network shows a high level of redundancy of histone modifications in differential modification profiles.

We found that different TFs have distinct target histone modification profiles. A histone modification high in one TF's profile could be low in another TF's profile (Figure 5a). We performed unsupervised clustering analysis for the histone modification profiles of all TFs, and detected two TF clusters (Figure 5a). One of the two clusters showed generally larger variations (more high and low signals) among histone modifications in the upstream region of their target genes, while the signals in the other cluster are more often around the mean (*P *< 10^-16^, *t*-test). We thus refer to the 68 TFs in the former cluster as 'histone modification-sensitive' (histone-sensitive) TFs, and the 135 TFs in the latter cluster as 'histone modification insensitive' (histone-insensitive) TFs (Additional file 6).

Correlations between pairs of histone modifications are shown in Figure 5c, based on their signals in the histone modification profiles for all TFs. Only pairs with strong correlation (r > 0.5 or < -0.5) were connected in the form of a correlation network. The dense connectivity in this network reveals strong pairwise redundancy of histone modification signals, which is also indicative of redundancy for predicting target genes.

We next examined the relative importance of each histone modification for predicting target genes of all TFs. Given a histone modification, we compared its signal difference between target and non-target genes of a TF. The signal difference is represented as t-statistics (see Materials and methods for details), which indicate the relative importance of that histone modification for predicting the target genes of a TF. A larger absolute value for a t-statistic indicates more importance. The t-statistics for all histone modifications form a TF-specific profile, denoted as differential modification profiles of the TF. Interestingly, histone-sensitive TFs and histone-insensitive TFs defined based on target histone modification profiles are also distinct according to their differential modification profiles (Figure 5b). This suggests that histone-sensitive and -insensitive TFs are actually robust clusters with different patterns of histone modifications in their target genes.

### Histone modification sensitivity of transcription factors

**Figure 6:**
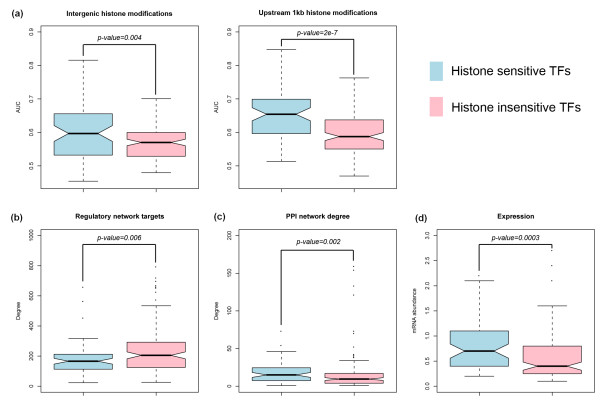
**Distinctions between histone-sensitive and -insensitive TFs**. **(a) **Target genes of histone-sensitive TFs are better predicted using intergenic or upstream histone modifications than are those of histone-insensitive TFs. **(b) **Histone-sensitive TFs have a smaller number of target genes in the regulatory network than histone-insensitive TFs. **(c) **Histone-sensitive TFs have a larger number of interaction partners in the protein-protein interaction (PPI) network. **(d) **Higher mRNA expression levels of histone-sensitive TFs.

To understand the biological nature of the histone-sensitive and -insensitive TFs, we explored the different features of these two TF classes under different biological 'contexts'. First, we observed a difference in predictive power of histone modifications for target gene prediction between the two TF classes. As shown in Figure 6a, histone modifications are generally more predictive of the target genes of histone-sensitive TFs than those of histone-insensitive TFs. This is due to the fact that target genes of histone-sensitive TFs have stronger histone modification signals, which substantially improve the performance of our chromatin model.

Histone-sensitive and -insensitive TFs also show distinct topological characteristics in biological networks. In general, histone-sensitive TFs have less target genes than histone-insensitive TFs (Figure 6b). Yu and Gerstein constructed a hierarchical network in yeast based on TF-TF regulation relationships identified by ChIP-chip [34]. We mapped the histone-sensitive and -insensitive TFs onto this hierarchical network and found that histone-sensitive TFs were enriched in the upper layers. This suggests that histone-sensitive TFs are more likely to act as 'managers' that regulate other TFs, while histone-insensitive TFs tend to be 'workers' at the bottom layer of the hierarchy (Table 2). We also examined the 'degrees' of these TFs in the protein-protein interaction networks [35]. Our results indicate that histone-sensitive TFs tend to have more physical interaction partners than histone-insensitive TFs (Figure 6c). The high connectivity of histone-sensitive TFs further implies their functional importance.

**Table 2:** Transcription factor histone sensitivity relates to hierarchical level in a regulatory network

Hierarchical levels in regulatory network	Number of histone-sensitive TFs	Number of histone-insensitive TFs
Bottom level		
Level 1	15	52
Upper levels		
Level 2	43	36
Level 3	6	8
Level 4	2	4
Total	51	48

Fisher exact test *P*-value = 0.0007 for separate levels; *P*-value = 0.0002 for combined upper levels.

Interestingly, the histone sensitivity of TFs also indicates distinct co-regulation relationships. Two TFs are said to be co-regulatory if their sets of targets significantly overlap. Among the approximately 14, 000 possible TF pairs, we found 1, 440 significant co-regulatory relationships (*P *< 0.05, Fisher's exact test). Among the TFs involved in co-regulatory relationships, 64 are histone sensitive and 95 are histone insensitive. Of the 1, 440 significant co-regulatory pairs, 447 are between two histone-sensitive TFs, 437 between two histone-insensitive TFs, and 556 between one histone-sensitive TF and one histone-insensitive TF. Fisher's exact test showed that histone-sensitive TFs are more likely to be involved in a co-regulatory relationship than histone-insensitive TFs (*P *< 10^-16^). In summary, the histone-sensitive TFs reside mostly in the upper layers of the regulatory network, and tend to work and communicate with other TFs during transcriptional regulation.

Furthermore, we found that the expression levels of histone-sensitive TFs were higher than those of the histone-insensitive TFs (Figure 6d). It seems that the histone sensitivity of TFs is also related to their biological functions. For example, TFs involved in cell cycle regulation predominantly belong to the histone-sensitive class (Table 3). Out of 17 cell cycle TFs reported in a previously study [36], only CST6 was classified to be histone insensitive. We also examined TFs that were specific to certain conditions - for example, heat shock or oxidative stress. Condition-specific TFs were classified into both histone-sensitive and -insensitive classes; thus, it is not obvious whether the histone sensitivity and condition specificity of TFs are related.

**Table 3:** Transcription factor histone sensitivity relates to cellular functions

Cellular functions	Histone-sensitive TFs	Histone-insensitive TFs
Cell cycle	ACE2, ASH1, CIN5, FKH1, FKH2, MBP1, MCM1, NDD1, RLM1, STB1, STE12, STP1, SWI4, SWI5, SWI6, TEC1	CST6
Heat shock or stress conditions	GAT1, MSN4, SKN7, YAP1	GLN3, HAL9, HMS2, HSF1, MGA1, MSN2, WAR1, USV1

### PSSM predictability and cooperativity of transcription factors

TFs exhibit different PSSM predictability in that the targets of some TFs are well predicted using the PSSM alone but others are not. PSSM predictability reflects the extent to which TFs recognize binding sites through motif matching. Since PSSMs are available for only 50 TFs, accounting for about a quarter of 203 TFs with histone-modification profiles, it is difficult to perform systematic identification and classification to categorize them as well-predictable or weakly predictable using PSSMs. However, we have identified ten TFs with a target prediction AUC of > 0.7 using their PSSMs alone, providing a confident subset of PSSM well-predictable TFs (Table 4). To check whether the high predictability is attributed to PSSM specificity, we calculated the information content of these PSSMs. We found that the information content of the ten well-predictable TFs' PSSMs is not different from those of other TFs (*P *= 0.4, Wilcoxon test). We investigated the expression level, number of target genes, and the hierarchy in the regulatory network of these ten TFs and found no significant difference from the other TFs. We will be able to make more confident conclusions when more PSSMs for TFs become available in the future.

**Table 4:** Top 10 PSSM well-predictable transcription factors

PSSM sensitive TFs	AUC
REB1	0.87
ABF1	0.86
CBF1	0.84
FHL1	0.83
RAP1	0.79
TYE7	0.77
SUM1	0.76
UME6	0.76
MBP1	0.72
GCN4	0.71

For TFs that are weakly predictable using PSSM information, we hypothesized that these TFs may bind to their targets indirectly by cooperating with other TFs. If this is the case, we would expect to predict the target genes of such a TF accurately by using the PSSM of its cooperative TF. We tested this by using each PSSM to predict targets of all TFs (Additional files 1 and 2). The targets of most TFs were best predicted by their own PSSMs, but some TFs have their targets better predicted using other TFs' PSSMs. For example, YAP1's target genes were better predicted using CAD1's PSSM - the AUC increased from 0.71 to 0.75. Similarly, the target genes of INO4 were better predicted using INO2's PSSM than it's own, with the AUC increasing from 0.78 to 0.81. In fact, YAP1 and CAD1 work together in stress-induced transcriptional responses, and INO2 and INO4 form heteromeric complexes involved in phospholipid biosynthesis [37,38]. We also found that the PSSMs of the cooperative TFs are actually quite similar, measured using a similarity score range from 0 to 1. The PSSMs of CAD1 and YAP1 have a similarity score of 0.72 (top 1% among all pairs), and those of INO2 and INO4 have a similarity score of 0.55 (top 5%). This further indicates the cooperation between the two TF pairs through indirect binding. Therefore, TF target gene prediction using 'cross-PSSMs' could help identify co-operative interactions between TFs. On the other hand, this suggests that using a TF's own PSSM may not always be best for predicting its target genes, especially when there is evidence it co-operates with another TF.

### Chromatin model improves prediction of TF binding sites

We have examined our chromatin model using the TF target data from large-scale ChIP-chip experiments [5], and shown its effectiveness for predicting target genes. The study by Harbison *et al*. [5] investigated TF binding within yeast promoter regions only. However, technical advancement of ChIP-chip and ChIP-seq has enabled us to obtain the binding sites of a TF across the whole genome. Given these more high-resolution data, our chromatin model can also be used to predict TFBSs. For demonstration, we use the ChIP-seq data for STE12 as an example [39].

**Figure 7:**
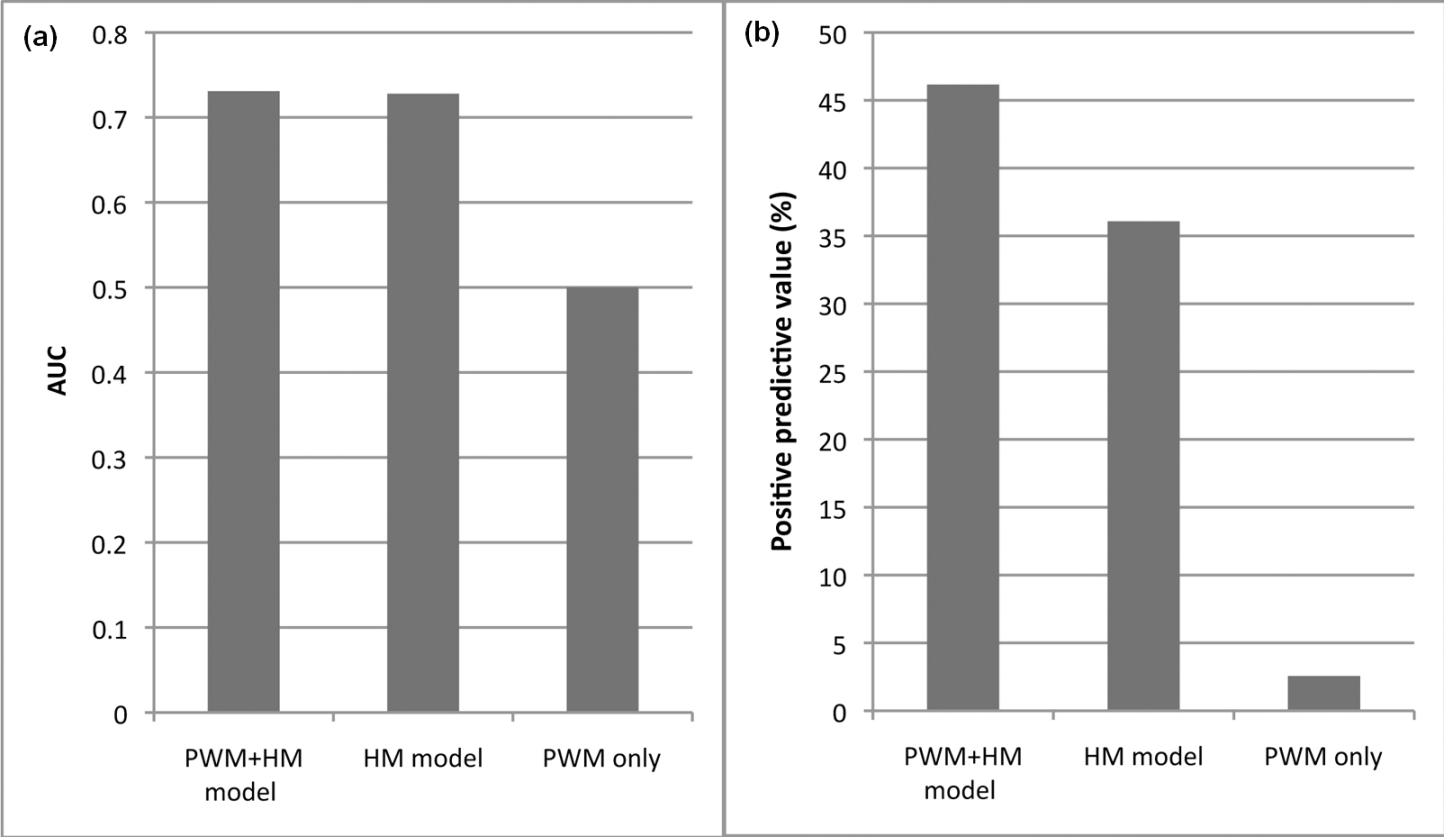
**Chromatin modifications improve STE12 binding site prediction**. **(a, b) **The AUC (a) and positive predictive value (b) of the histone-only model (HM), the PSSM-only model (PWM) and the combined model (PWM+ HM) for STE12 binding site prediction.

We examined chromatin signals around STE12 binding peaks. We found many histone marks, such as H3K9ac, were enriched in the peak regions (Additional file 7), implying that they are informative for predicting TFBSs. We separated the yeast genome into 100-nucleotide bins, and divided them into positive bins and negative bins according to their overlap with STE12 binding peaks. Then we constructed SVM classification models (histone modification model, PSSM model and combined model) to predict positive bins in a similar way as predicting target genes. We estimated their prediction accuracy by using a cross-validation method, which verified the effectiveness of the chromatin model for binding site prediction (Figure 7). The prediction power of using both histone modification and PSSM information is roughly the same as when using only histone modification (AUC = 0.73), whereas using PSSM only is close to random (AUC = 0.5). The advantage of combining histone modifications with PSSM information, however, is clearly demonstrated when the positive predictive value (PPV; the fraction of positive predictions being true positives) is concerned (Figure 7). This is of particular importance in TFBS prediction because reducing false positive predictions is the major challenge of model improvement.

### Comparison with previous methods

We compared our SVM-based method with several previously published approaches, including Cluster-Buster [13], MCAST [40], EEL [41], and Stubb [16]. We calculated the prediction accuracy of each method by applying it to 10 TFs with more than 200 target genes under the YPD condition. As shown in Table 5, our method integrating histone modification and PSSM data sets achieves the best prediction for most TFs. For histone-senstive TFs such as SWI4 and SWI6, including histone modification data can improve target prediction accuracy substantially, and PSSM alone gives relatively poor predictions no matter what algorithms are used to search for TFBSs.

**Table 5:** Comparison of several computational methods for target gene prediction

	ROC AUC							
	
	Number of target genes	HIS+PSSM	HIS alone	PSSM alone (FIMO)	Cluster-Buster	MCAST	EEL	Stubb
ABF1	549	0.830	0.736	0.781	0.776	0.676	0.807	0.893
FHL1	207	0.957	0.963	0.827	0.855	0.874	0.852	0.887
FKH1	284	0.656	0.625	0.606	0.680	0.546	0.661	0.725
FKH2	216	0.723	0.694	0.664	0.698	0.566	0.688	0.735
HAP1	215	0.738	0.711	0.635	0.675	0.624	0.676	0.663
RAP1	408	0.865	0.818	0.805	0.752	0.774	0.802	0.811
REB1	278	0.773	0.623	0.774	0.727	0.818	0.765	0.758
SWI4	252	0.831	0.790	0.634	0.680	0.626	0.664	0.651
SWI6	230	0.809	0.768	0.719	0.720	0.629	0.742	0.665
UME6	298	0.854	0.767	0.831	0.774	0.783	0.814	0.815

HIS, histone modification-based method.

Among the previously published methods, EEL and Stubb take advantage of conservation of TF binding motifs between related species, and as shown they achieve relatively more accurate prediction results than FIMO, Cluster-Buster and MCAST. We also tried the 'Chromia' method proposed by Won *et al*. [24]. Similar to our method, Chromia integrates histone modification and PSSM data sets but using a hidden Markov model. The method has shown impressive performance when applied to genome-wide ChIP-seq data in mouse for predicting TFBSs. However, when applied to the yeast data in our case, it does not result in good prediction due to the low coverage of the histone modification and TF binding data sets [5,23]. For example, the arrays used for the Pokholok *et al*. ChIP-chip data contain approximately 42, 000 probes (60-mers), representing only about 20% of the yeast genome [23]. The arrays used for identifying yeast TFBSs are essentially promoter arrays, covering only DNA regions around the transcription start site of yeast ORFs [5]. In practice, our method requires only data for interested regions (for example, promoter regions), and thereby is more flexible and can be applied to a wide range of data sets.

We examine the effectiveness of these methods for predicting STE12 binding sites (see 'Chromatin model improves prediction of TFBSs' section). Chromia predicts STE12 binding sites with an AUC of 0.66 and a PPV of 5.6%, presumably also due to the low resolution of the histone modification data. Those motif-based methods perform similarly to our PSSM-based methods (Figure 7), and taking into account conservation does not lead to significant improvement for the STE12 case.

## Discussion

### Histone-sensitive and -insensitive transcription factors

We classified the 203 yeast TFs used in our study into 68 histone-sensitive and 135 histone-insensitive TFs based on the upstream histone modification signals of their target genes. The two classes have generally opposite characteristics with regard to histone modification signals, expression levels, topology in regulatory networks and other biological features.

Steinfeld *et al*. [42] have discovered a list of TF-chromatin modifier interactions in yeast from genome-wide analysis of high-throughput experiments. Among the 35 TFs that interact with chromatin modifiers, 20 are classified as histone-sensitive and 15 as histone-insensitive according to our analysis. Namely, there is a significant enrichment of histone-sensitive TFs (*P *= 0.001, Fisher's exact test) in the chromatin modifier interacting TFs. This suggests that histone-sensitive TFs are more likely to interact with chromatin modifiers, consistent with the observation that their target genes tend to have stronger histone modification signals.

Hitone-sensitive TFs might target highly regulated genes. It is known that gene expression is regulated by specific TFs and their orchestrating chromatin modification enzymes. Thus, stronger histone modification signals upstream of the target genes of the histone-sensitive TFs might imply more intensive transcriptional regulation. Our results showed that cell cycle TFs were mostly histone-sensitive TFs, consistent with the previous knowledge that the cell cycle is highly regulated to achieve cyclical expression of genes.

Most histone modification data used in this study are derived from yeast grown on YPD medium. We found that histone-sensitive TFs tend to be active under the YPD medium condition, as indicated by a larger number of target genes and higher expression levels with respect to those insensitive TFs. It is possible that histones upstream of the target genes of the histone-sensitive TFs have more chance to be modified by histone modification enzymes because they are recruited by these more active TFs.

We found that histone-sensitive TFs were enriched in higher layers of the hierarchical regulatory network. This suggests that histone-sensitive TFs tend to be 'managers' that regulate other TFs and, for such a reason, their binding to target genes is highly regulated through histone modifications. Consistent with this hypothesis, we have observed stronger histone modification signals in the upstream regions of histone-sensitive TFs' target genes. We caution here that the classification of the histone-sensitive and -insensitive TFs is based only on the histone modification signals of their target genes and the contribution of these signals to target predictions. Further experiments might be worthwhile to investigate the relationship between the target selection of the TF classes and histone modifications in more detail.

A more recent paper in human showed the capability of using unique chromatin signatures to identify two distinct classes of genomic elements, active and poised enhancers [19]. Consistently, here we find in yeast that the histone modifications are also informative for distinguishing ChIP-chip verified and non-verified binding sites. As we demonstrated, the method could be applied to identify target genes and the genome-wide binding sites of TFs.

### Contribution of histone modifications to condition-specific target prediction

It is widely known that transcriptional regulation is condition specific in that TFs change their binding sites under different conditions. We show here that histone modification data are most predictive of TF target binding under the same condition. This is true especially for those TFs that are mostly active in specific stress conditions.

Because of limited resources, it is impossible to perform exhaustive experiments for every TF, cell type, and species and all possible conditions. Meanwhile, the TF binding recognition motifs, PSSMs, are generally thought to be non-condition-specific, not changing under different conditions [3]. Thus, prediction based on motif information alone cannot provide condition-specific target genes for a TF. As an alternative method, we propose the feasibility of predicting target genes of a TF under a condition of interest by combining histone modification data under that condition with its PSSM. In this way, we can achieve much higher results than using PSSM alone. More importantly, since histone modifications reflect the chromatin states in a specific condition or tissue, the predicted targets are also condition and tissue specific.

### Combinatorial interaction of TFs: direct and indirect binding

When a TF binds directly to the promoter regions of its target genes, the enriched motifs identified from its binding sites can be regarded as its own PSSM. However, TFs do not always act individually; sometimes they cooperate with (physically bind to) each other to form regulatory functional units, such as the yeast cell cycle complexes SBF (SWI4-SWI6) and MBF (MBP1-SWI6) [30]. In these indirect binding cases, it is important to distinguish the TFs that are motif-recognizing and those that are not.

By examining the PSSM sensitivity of TFs, we were able to infer some possible combinatorial interactions between TFs. If TF A's targets are better predicted by using TF B's PSSM instead of its own PSSM, then this is an indication of potential cooperation between the two TFs. In particular, TF B directly binds to promoter regions through its PSSM, and TF A indirectly binds to promoter regions through physical binding to TF B [43]. This is also referred to as indirect piggy-back binding [6].

PSSM sensitivity under indirect TF binding is important for our target gene prediction model. Instead of using a TF's own PSSM, the PSSM of another TF through which the TF binds should be used for more accurate predictions. Therefore, identifying those cases before using our model will be necessary to achieve better results.

### Implications on gene expression regulation

We show in this study that incorporating chromatin modification information could substantially improve the prediction of TF target genes. In fact, chromatin modifications relate to gene expression regulation on two levels [3]. First, chromatin is modified to form euchromatin, within which genes can be turned on and off, or heterochromatin, within which genes are silenced. Second, euchromatin is further modified by enzymes recruited by specific TFs to mark the 'on and off' status of transcription. We examined target versus non-target differential histone modification profiles for each individual TF, and observed TF-specific chromatin modifications marked in the target genes. Therefore, we suggest that chromatin modifications might function as both non-specific euchromatin marks and TF-specific regulatory marks. Our model takes advantage of the chromatin information from both of these levels.

However, the sequential order of chromatin modification and TF binding, in terms of time and causality, is still not quite clear. It is possible that one of them happens first and then drives the occurrence of the other. The other possibility is that the two events might interact in a feedback manner to regulate gene expression. More fine-tuned experiments in the future would be helpful for unraveling the time-dependent interaction between chromatin modification and TF binding.

## Materials and methods

### Chromatin modification data

The yeast histone modification data sets used in this study are basically from two sources. The first data set is from Pokholok *et al*. [23] and contains the profiles of 14 chromatin features under YPD or H_2_O_2 _conditions. These chromatin features include histone H3 and H4 occupation, H3K9ac, H3K14ac, H4K5ac8ac12ac16ac, H3K4me1, H3K4me2, H3K4me3, H3K36me3, and H3K79me3. The profiles of these features were measured by ChIP-chip experiments using over 40, 000 probes, which cover 85% of the yeast genome. We calculated the signal of each chromatin feature in the 1-kb upstream region of each ORF by averaging signals of all the probes within this region. Similarly, for each ORF the average signal of each feature in the 1-kb region downstream of the start codon was also calculated. We named these the upstream chromatin signal and downstream chromatin signal for ORFs, respectively.

The second data set is from Kurdistani *et al*. [22]. These data contain levels of acetylation of 11 lysines in intergenic regions (IRs) as well as ORF regions. These profiles were also measured using ChIP-chip experiments. These 11 histone acetylations are H2AK7ac, H2BK11ac, H2bK16ac, H3K9ac, H3K14ac, H3K18ac, H3K23ac, H3K27ac, H4K8ac, H4K12ac and H4K16ac. We named the signals in IRs and ORFs as the IR chromatin signal and coding region chromatin signal for ORFs, respectively.

### Target genes of yeast transcription factors

Target genes for 203 yeast TFs under various conditions (including YPD and H_2_O_2_) were identified using the ChIP-chip experiments by Harbison *et al*. [5]. For each binding interaction, a probability score (*P*-value) was calculated, measuring the binding potential of a TF with the promoter region of a gene.

When TF target genes are determined according to ChIP-chip data, one needs to set a cutoff for *P*-values, which indicates the confidence of regulation of genes by TFs. A small (strict) *P*-value cutoff would result in fewer but more confident target genes, while a large (loose) *P*-value cutoff would do the opposite. For instance, there are 159 target genes for RAP1 using a cutoff value of 0.001, while the target gene number increases to 581 when a cutoff value of 0.05 is used. We therefore tested the influence of the *P*-value cutoff on our model performance. As shown in Figure 2a, our results indicate that a more stringent *P*-value cutoff (that is, a smaller target gene set) improves the prediction accuracy of our model. Moreover, at all cutoff values the models combining histone modification and PSSM data outperform the models using either of them alone. On the other hand, a more stringent cutoff results in less target genes. To ensure enough positive target genes for model training, we decided to use 0.01 as the *P*-value cutoff in our analysis.

### Position-specific scoring matrices of transcription factors

Two sets of PSSMs for yeast TFs have been identified previously using different strategies [10,18]. The first set was downloaded from [20], which was based on *de novo *motif finding in all yeast promoter sequences. The promoter DNA sequences (from the start codon of an ORF to 800 bp upstream) of all yeast ORFs were analyzed to identify enriched motifs using the AlignACE program [44]. A total of 666 motifs were found, among which 51 can be associated with known yeast TFs. The occurrences and matching scores of these motifs in the promoter regions of all yeast genes were also provided by Beer and Tavazoie [20].

The second set of PSSMs was from [5], which is based on motif analysis of target promoters identified by the ChIP-chip experiment. Details on the motif discovery procedure can be found in [5]. In brief, motifs for a TF were discovered by applying a suite of motif discovery programs to the intergenic sequences identified by the binding data for this factor. The resulting motifs were subsequently clustered, filtered and selected to give rise to a single PSSM that can best represent the motif of a factor. For some factors the above procedure failed to identify their motifs and in such cases motifs were derived from the literature or databases.

The information content (IC) of a PSSM is calculated as:

$$IC=-\sum_{i,j}p_{i,j}\times \log\left(p_{i,j}∕p_{b}\right)$$

where *i *and *j *represent positions in PSSMs and four nucleotides, respectively. *p_i, j _*is then the weight at each PSSM position of each nucleotide, and *p_b _*is the background nucleotide frequency of the *S. cerevisiae *genome. Specifically, we use 37% as the GC content to calculate *p_b _*for each nucleotide.

The similarity between two PSSMs is calculated as the averaged dot product at each PSSM position:

$$Similarity=\frac{1}{n}\sum_{i,j}p_{1,i,j}\times p_{2,i,j}$$

where *n *is the length of PSSMs. If two PSSMs are of different lengths, we compare each possible alignment of the two PSSMs with no gap, and keep the maximum similarity from each alignment. The similarity score is in the range 0 to 1.

### Searching promoters for known motifs

Given the list of PSSMs for TFs, we searched the promoters of all yeast genes for occurrences of these motifs using FIMO of the MEME suite [45]. The promoter region was defined as the DNA region from the start codon to 800 bp upstream of an ORF. The cumulative matching score of the occurrences of a motif in the promoter region of a gene was calculated, which was subsequently used as a feature for predicting TF target genes.

### Comparison of chromatin modifications between ChIP-chip-verified TFBSs and non-verified motif matching sites

We performed comparative analysis of chromatin modification differences between TFBSs verified by ChIP-chip experiments and those non-verified motif matching sites for TFs with available PSSMs. SWI4 is discussed here as an example. A list of binding sites (TFBSs) of the factor SWI4 was downloaded from the Saccharomyces Genome Database [46]. This list contains 99 binding sites that are targeted by SWI4 in YPD medium according to the ChIP-chip data. We also collected a list of non-TFBSs by selecting DNA regions that are consistent with the SWI4 motif but not targeted by SWI4 in YPD medium as indicated by the ChIP-chip results (*P *> 0.4). These non-TFBSs were further filtered to ensure that there is no SWI4 TFBSs within the nearby 2-kb region, which ultimately resulted in 485 non-TFBSs for SWI4. All of the TFBSs and non-TFBSs are less than 20 bp in size. The levels of chromatin features in these TFBSs and non-TFBSs were calculated based on the intensities of probes covering them. Finally, the signal of the 14 chromatin features was compared between the TFBS and non-TFBS groups using the *t*-test.

### Support vector machine model for transcription factor target prediction

For a TF in each gene we obtained the following features: cumulative matching score from motif searching, the upstream and downstream signal of 14 histone or histone modification profiles, and the IR and coding region signal of 11 histone acetylation profiles. Based on the ChIP-chip data from [5], genes were classified into target and non-target sets for a TF. All or subsets of these features were integrated using a SVM model [26] for predicting target genes of a TF. The radial kernel was used for training and predicting in the SVM classification model.

We evaluated the performances of the models using two-fold cross-validation. Specifically, we randomly split the data into two sets of equal sizes, a training set and a testing set. The model was then trained using the training set and applied to the testing set to predict target genes. The prediction power of the model was estimated based on the testing set. In general, the SVM model outputs a probability indicating how likely a gene is to be the target of a TF. By setting different cutoff values, we can balance the sensitivity (true positive rate) and specificity (true negative rate) of predictions of the model. The plot of the sensitivity versus '1 - specificity' is called the ROC, which can be used to show the classification accuracy of the SVM model. The AUC can be used to summarize the prediction power of the model. For each TF, we repeated this process 50 times and the average of the AUC values was calculated to represent the prediction accuracy.

Based on the above-described SVM classification method, we constructed and compared several different models for TF target prediction, each taking advantage of different features (histone modifications, PSSM information, gene expression levels) or a combination of these. First, we prepared several groups of histone modification features. Based on the Pokholok *et al*. data [23], we calculated the signals of 14 types of histone modification in the upstream (500 bp, 1, 000 bp) and the downstream (500 bp, 1, 000 bp) regions of the transcription start sites of all yeast genes. From the Kurdistani *et al*. data [22], we collected the signals of 11 types of histone modification in the intergenic and ORF regions. Second, we calculated the cumulative matching score of the occurrences of the PSSM of each TF in the promoter region of genes, resulting in the PSSM features. Third, these features together with the expression levels of genes were selected and used as the predictors to classify targets versus non-targets of TFs. For instance, in the histone + PSSM model for factor RAP1, the histone modification features and the PSSM feature of the RAP1 motif were selected as the predictors of the SVM. In 'Results', we mostly used the 1, 000-bp upstream and the 1, 000-bp downstream histone modification features as predictors, but the other groups of histone modification features were also investigated for comparison purposes. The SVM function in the R package 'e1071' was utilized to implement the models with default parameter settings. For the SVM the radial basis function kernel was used since it achieved the best performance according to the cross-validation results.

### Clustering of TFs using target chromatin modification profile

For each TF, a target histone modification profile was calculated by averaging histone modification signals among all its targets. The 1-kb upstream chromatin modifications from Pokholok *et al*. [20] and intergenic chromatin modifications from Kurdistani *et al*. [22] were used. An unsupervised k-means clustering algorithm was performed to generate two TF clusters, histone-sensitive and -insensitive TFs, using their target histone modification profiles.

To understand the relative importance of each chromatin modification to target prediction, target-non-target differential histone modification profiles for TFs were calculated based on t-statistics. For each chromatin modification in a differential modification profile for a TF, modification signals for target genes and non-target genes were collected and a t-statistic calculated. The t-statistics in differential modification profiles indicated the directional significance of chromatin modifications to distinguish target genes.

### Inferring interactions between transcription factors

The target genes identified by ChIP-chip experiments could be either direct or indirect targets of a TF. For example, if two TFs, A and B, interacte with each other, the ChIP-chip for A can potentially identify target genes of B as well. Conversely, the existence of TF B's motif would be informative for predicting target genes of TF A. We used the TF target prediction model with chromatin modifications and TFs' own PSSMs, and then compared the model's AUC values to those derived using models with chromatin modifications and other TFs' PSSMs. Models with improved AUC performances suggest better predictive power with PSSMs other than a TF's own PSSM. These cases might indicate interactions between TFs.

### Application of previously reported methods

We ran all methods with their default parameter settings. Internal thresholding was turned off in all cases to report a full list of predictions with scores. PSSMs of ten TFs and upstream 1-kb DNA sequences for all annotated yeast *S. cerevisiae *ORFs were used as inputs to MCAST [22] and Cluster-Buster [22]. Pairwise pre-aligned upstream 1-kb DNA sequences of all annotated *S. cerevisiae *and *Saccharomyces paradoxus *orthologous ORFs were used instead for running EEL [22] and Stubb [22]. Predicted binding targets with respective scoring systems from the programs were collected for all ten TFs. ROC curves and AUCs were calculated based on the same thresholding scheme for all methods.

## Abbreviations

AUC: area under ROC curve; bp: base pair; ChIP-chip: chromatin immunoprecipitation with microarray hybridization; ChIP-seq: chromatin immunoprecipitation with massively parallel DNA sequencing; IR: intergenic region; ORF: open reading frame; PPV: positive predictive value; PSSM: position-specific scoring matrix; ROC: receiver operator characteristic; SVM: support vector machine; TF: transcription factor; TFBS: transcription factor binding site.

## Authors' contributions

CC conceived of the study and participated in the analysis. CS participated in the analysis. KY participated in the data analysis. MG participated in its design and coordination. All authors read and approved the final manuscript.

## Supplementary Material

Additional file 1 **Table S1**. Click here for file

Additional file 2 **Table S2**. Click here for file

Additional file 3 **Table S3**. Click here for file

Additional file 4 **Table S4**. Click here for file

Additional file 5 **Table S5**. Click here for file

Additional file 6 **Table S6**. Click here for file

Additional file 7 **Figure S1**. Click here for file
