# Supplementary material for: Genome-wide analysis of chromatin features identifies histone modification sensitive and insensitive yeast transcription factors
Source: Genome Biol. 2011 Nov 7;12(11):R111. doi: 10.1186/gb-2011-12-11-r111 (PMC3334597; doi:10.1186/gb-2011-12-11-r111)
Supplement: Additional file 1 — Table S1. [file gb-2011-12-11-r111-S1.DOC]

**Table S1:** AUC of PSSM, Histone and Histone+PSSM models using *Beer et al* PSSMs

| **TF** | **Histone+PSSM** | **Histone** | **PSSM** |
| --- | --- | --- | --- |
| ABF1 | 0.89 | 0.72 | 0.87 |
| ACE2 | 0.73 | 0.72 | 0.54 |
| CBF1 | 0.87 | 0.67 | 0.84 |
| CIN5 | 0.80 | 0.75 | 0.64 |
| FKH1 | 0.65 | 0.63 | 0.59 |
| GCN4 | 0.74 | 0.58 | 0.70 |
| HAP4 | 0.77 | 0.74 | 0.65 |
| INO4 | 0.76 | 0.74 | 0.62 |
| MAC1 | 0.63 | 0.61 | 0.51 |
| MBP1 | 0.72 | 0.68 | 0.62 |
| MCM1 | 0.80 | 0.69 | 0.70 |
| MSN4 | 0.59 | 0.61 | 0.52 |
| MSN4 | 0.63 | 0.63 | 0.51 |
| NRG1 | 0.75 | 0.74 | 0.57 |
| OAF1 | 0.65 | 0.61 | 0.49 |
| PHO4 | 0.51 | 0.49 | 0.50 |
| RAP1 | 0.88 | 0.82 | 0.78 |
| REB1 | 0.87 | 0.62 | 0.87 |
| RFX1 | 0.60 | 0.53 | 0.55 |
| RPN4 | 0.63 | 0.59 | 0.50 |
| SKN7 | 0.84 | 0.84 | 0.62 |
| STE12 | 0.76 | 0.66 | 0.65 |
| SUM1 | 0.89 | 0.86 | 0.78 |
| SWI4 | 0.84 | 0.80 | 0.65 |
| UME6 | 0.84 | 0.75 | 0.76 |
| YAP1 | 0.70 | 0.62 | 0.62 |
