# Supplementary material for: Genome-wide analysis of chromatin features identifies histone modification sensitive and insensitive yeast transcription factors
Source: Genome Biol. 2011 Nov 7;12(11):R111. doi: 10.1186/gb-2011-12-11-r111 (PMC3334597; doi:10.1186/gb-2011-12-11-r111)
Supplement: Additional file 2 — Table S2. [file gb-2011-12-11-r111-S2.DOC]

**Table S2:** AUC of PSSM, Histone and Histone+PSSM models using *Frankel et al* PSSMs

| **TF** | **Histone+PSSM** | **Histone** | **PSSM** |
| --- | --- | --- | --- |
| ABF1 | 0.83 | 0.74 | 0.78 |
| ACE2 | 0.72 | 0.72 | 0.54 |
| DIG1 | 0.69 | 0.60 | 0.55 |
| FHL1 | 0.96 | 0.96 | 0.83 |
| FKH1 | 0.66 | 0.62 | 0.61 |
| FKH2 | 0.72 | 0.69 | 0.66 |
| HAP1 | 0.74 | 0.71 | 0.63 |
| HAP4 | 0.73 | 0.70 | 0.50 |
| INO2 | 0.76 | 0.61 | 0.69 |
| INO4 | 0.80 | 0.73 | 0.61 |
| NDD1 | 0.77 | 0.73 | 0.62 |
| PDR1 | 0.73 | 0.75 | 0.50 |
| RAP1 | 0.87 | 0.82 | 0.81 |
| REB1 | 0.77 | 0.62 | 0.77 |
| RFX1 | 0.66 | 0.57 | 0.54 |
| STB4 | 0.71 | 0.68 | 0.52 |
| STB5 | 0.66 | 0.55 | 0.53 |
| SUM1 | 0.89 | 0.87 | 0.64 |
| SUT1 | 0.80 | 0.80 | 0.62 |
| SWI4 | 0.83 | 0.79 | 0.63 |
| SWI6 | 0.81 | 0.77 | 0.72 |
| TEC1 | 0.72 | 0.64 | 0.50 |
| TYE7 | 0.78 | 0.50 | 0.76 |
| UME6 | 0.85 | 0.77 | 0.83 |
