# Supplementary material for: Genome-wide analysis of chromatin features identifies histone modification sensitive and insensitive yeast transcription factors
Source: Genome Biol. 2011 Nov 7;12(11):R111. doi: 10.1186/gb-2011-12-11-r111 (PMC3334597; doi:10.1186/gb-2011-12-11-r111)
Supplement: Additional file 3 — Table S3. [file gb-2011-12-11-r111-S3.DOC]

**Table S3:** Number of genes with PSSM occurrences and AUC of PSSM (AUC_P), Histone (AUC_H) and Histone+PSSM (AUC_HP) models using *Beer et al* PSSMs

| **Number of genes with PSSM** | **PSSM** | **Number of target genes** | **AUC_HP** | **AUC_H** | **AUC_P** |
| --- | --- | --- | --- | --- | --- |
| 292 | STE12 | 130 | 0.7625 | 0.6557 | 0.649 |
| 461 | NRG1 | 136 | 0.7534 | 0.7374 | 0.5722 |
| 493 | FKH1 | 284 | 0.6543 | 0.6303 | 0.5859 |
| 497 | INO4 | 194 | 0.7608 | 0.7354 | 0.6192 |
| 548 | OAF1 | 169 | 0.6547 | 0.6059 | 0.4909 |
| 555 | CIN5 | 274 | 0.7975 | 0.7533 | 0.6449 |
| 572 | PHO4 | 165 | 0.5112 | 0.4874 | 0.4972 |
| 585 | REB1 | 278 | 0.8663 | 0.6238 | 0.8707 |
| 665 | MCM1 | 163 | 0.8038 | 0.6943 | 0.6951 |
| 667 | RPN4 | 176 | 0.6266 | 0.5925 | 0.496 |
| 697 | YAP1 | 156 | 0.7048 | 0.6237 | 0.6166 |
| 710 | UME6 | 298 | 0.8353 | 0.7509 | 0.7593 |
| 726 | CBF1 | 112 | 0.8742 | 0.6725 | 0.8412 |
| 749 | MAC1 | 134 | 0.6271 | 0.6123 | 0.5109 |
| 763 | ACE2 | 145 | 0.7293 | 0.7191 | 0.5354 |
| 767 | GCN4 | 143 | 0.7437 | 0.584 | 0.7001 |
| 871 | MBP1 | 229 | 0.7207 | 0.6819 | 0.625 |
| 905 | SKN7 | 166 | 0.8389 | 0.842 | 0.6242 |
| 938 | ABF1 | 549 | 0.8935 | 0.7247 | 0.8662 |
| 953 | SUM1 | 121 | 0.8856 | 0.8571 | 0.7766 |
| 983 | HAP4 | 126 | 0.7742 | 0.7371 | 0.6505 |
| 1030 | RFX1 | 105 | 0.6027 | 0.5344 | 0.5467 |
| 1191 | SWI4 | 252 | 0.8405 | 0.7982 | 0.6481 |
| 1217 | MSN4 | 145 | 0.5904 | 0.6145 | 0.5167 |
| 1953 | RAP1 | 408 | 0.876 | 0.8226 | 0.7815 |
