# Supplementary material for: Genome-wide analysis of chromatin features identifies histone modification sensitive and insensitive yeast transcription factors
Source: Genome Biol. 2011 Nov 7;12(11):R111. doi: 10.1186/gb-2011-12-11-r111 (PMC3334597; doi:10.1186/gb-2011-12-11-r111)
Supplement: Additional file 4 — Table S4. [file gb-2011-12-11-r111-S4.DOC]

**Table S4:** Number of genes with PSSM occurrences and AUC of PSSM (AUC_P), Histone (AUC_H) and Histone+PSSM (AUC_HP) models using *Frankel et al* PSSMs

| **Number of genes with PSSM** | **PSSM** | **Number of target genes** | **AUC_HP** | **AUC_H** | **AUC_P** |
| --- | --- | --- | --- | --- | --- |
| 79 | GCN4_SM | 143 | 0.7579 | 0.5359 | 0.741 |
| 957 | YAP6 | 167 | 0.83 | 0.8162 | 0.499 |
| 1020 | CBF1_SM | 112 | 0.8938 | 0.6733 | 0.9016 |
| 1088 | MCM1_ALPHA | 163 | 0.7848 | 0.6809 | 0.6897 |
| 1151 | TYE7_YPD | 105 | 0.7825 | 0.4972 | 0.7604 |
| 1579 | ADR1 | 146 | 0.6688 | 0.6787 | 0.4992 |
| 1958 | PDR1_YPD | 163 | 0.7297 | 0.7479 | 0.5002 |
| 2024 | RPN4_H2O2LO | 176 | 0.6104 | 0.6008 | 0.4992 |
| 2124 | RLM1 | 120 | 0.7957 | 0.7715 | 0.5239 |
| 2463 | MBP1_H2O2HI | 229 | 0.7678 | 0.6594 | 0.7193 |
| 2743 | UME6_YPD | 298 | 0.8538 | 0.7674 | 0.8311 |
| 2744 | FKH1_YPD | 284 | 0.6565 | 0.6246 | 0.6064 |
| 2959 | SUT1_YPD | 172 | 0.795 | 0.8039 | 0.6166 |
| 3040 | SWI6_YPD | 230 | 0.809 | 0.7681 | 0.7189 |
| 3124 | PHD1_BUT90 | 166 | 0.7572 | 0.7614 | 0.5264 |
| 3139 | YAP5 | 167 | 0.7788 | 0.7746 | 0.5 |
| 3218 | AFT2_H2O2LO | 112 | 0.5253 | 0.5792 | 0.4939 |
| 3220 | YAP1_H2O2LO | 156 | 0.677 | 0.6283 | 0.5281 |
| 3221 | SWI5 | 201 | 0.6367 | 0.629 | 0.5247 |
| 3423 | CIN5_H2O2LO | 274 | 0.7998 | 0.7613 | 0.6134 |
| 3423 | NDD1_YPD | 190 | 0.7674 | 0.7334 | 0.623 |
| 3477 | MSN4 | 145 | 0.6167 | 0.6496 | 0.5203 |
| 3498 | SUM1_YPD | 121 | 0.891 | 0.8697 | 0.6356 |
| 3560 | STB5_YPD | 105 | 0.6588 | 0.5493 | 0.5264 |
| 3683 | DIG1_YPD | 110 | 0.6926 | 0.5979 | 0.5467 |
| 3838 | REB1_YPD | 278 | 0.7732 | 0.6227 | 0.7738 |
| 3862 | SWI4_YPD | 252 | 0.8311 | 0.79 | 0.6336 |
| 4014 | PHO2_H2O2HI | 141 | 0.5874 | 0.6138 | 0.51 |
| 4017 | YOX1 | 111 | 0.7199 | 0.7117 | 0.5091 |
| 4068 | FKH2_YPD | 216 | 0.7227 | 0.6939 | 0.664 |
| 4094 | STE12_ALPHA | 130 | 0.7001 | 0.6617 | 0.5809 |
| 4098 | INO2_YPD | 114 | 0.7642 | 0.6052 | 0.6852 |
| 4149 | HAP1_YPD | 215 | 0.7376 | 0.7108 | 0.6348 |
| 4343 | SMP1 | 181 | 0.6923 | 0.7046 | 0.4893 |
| 4343 | STB4_YPD | 117 | 0.7054 | 0.683 | 0.5185 |
| 4412 | TEC1_YPD | 114 | 0.7248 | 0.6351 | 0.4952 |
| 4434 | PHO4_PI. | 165 | 0.5002 | 0.5073 | 0.4929 |
| 4471 | SKN7_H2O2LO | 166 | 0.8452 | 0.826 | 0.6874 |
| 4622 | FHL1_YPD | 207 | 0.9567 | 0.9631 | 0.8272 |
| 4664 | RFX1_YPD | 105 | 0.6617 | 0.574 | 0.5396 |
| 4687 | ACE2_YPD | 145 | 0.7245 | 0.7193 | 0.5375 |
| 4753 | ABF1_YPD | 549 | 0.8299 | 0.7363 | 0.781 |
| 4768 | ROX1 | 172 | 0.8145 | 0.8125 | 0.5131 |
| 4988 | NRG1_H2O2HI | 136 | 0.7548 | 0.7408 | 0.4907 |
| 5211 | MET32 | 154 | 0.6171 | 0.6058 | 0.5002 |
| 5504 | HAP4_YPD | 126 | 0.7337 | 0.7012 | 0.5001 |
| 5506 | RAP1_YPD | 408 | 0.8652 | 0.8176 | 0.8053 |
| 5550 | INO4_YPD | 194 | 0.7961 | 0.7318 | 0.611 |
| 5727 | MAC1 | 134 | 0.6487 | 0.6375 | 0.4937 |
| 5810 | DAL82_SM | 103 | 0.4956 | 0.5296 | 0.4884 |
