# Supplementary material for: Genome-wide analysis of chromatin features identifies histone modification sensitive and insensitive yeast transcription factors
Source: Genome Biol. 2011 Nov 7;12(11):R111. doi: 10.1186/gb-2011-12-11-r111 (PMC3334597; doi:10.1186/gb-2011-12-11-r111)
Supplement: Additional file 5 — Table S5. [file gb-2011-12-11-r111-S5.DOC]

**Table S5:** Number of target genes and prediction accuracy by the Expression only model, the Histone+PSSM+Expression model and the PSSM+Expression model. The predicted accuracy was represented as the AUC value, for which the mean and standard deviation were calculated based on 50 validations. In each validation, one half randomly selected genes were used as the training set and the remaining were used as the testing set. The target gene numbers were determined based on ChIP-Chip data (P<0.01). PSSMs from Beer et al. were used. Note that the results are similar to Table S1 and that the contribution of gene expression level is small.

| **TF** | **Number of target genes** | **Expression Model (AUC)** | | **Histone + PSSM + Expression Model (AUC)** | | **PSSM + Expression Model (AUC)** | |
| --- | --- | --- | --- | --- | --- | --- | --- |
|  |  | **MEAN** | **STD** | **MEAN** | **STD** | **MEAN** | **STD** |
| ABF1 | 549 | 0.58 | 0.06 | 0.90 | 0.01 | 0.88 | 0.01 |
| ACE2 | 145 | 0.52 | 0.02 | 0.73 | 0.02 | 0.53 | 0.04 |
| ADR1 | 146 | 0.54 | 0.05 |  |  |  |  |
| AFT2 | 112 | 0.51 | 0.03 |  |  |  |  |
| CBF1 | 112 | 0.49 | 0.02 | 0.87 | 0.03 | 0.84 | 0.03 |
| CIN5 | 274 | 0.52 | 0.03 | 0.80 | 0.02 | 0.67 | 0.03 |
| CST6 | 106 | 0.5 | 0.02 |  |  |  |  |
| DAL82 | 103 | 0.52 | 0.03 |  |  |  |  |
| DIG1 | 110 | 0.5 | 0.01 |  |  |  |  |
| FHL1 | 207 | 0.79 | 0.01 |  |  |  |  |
| FKH1 | 284 | 0.5 | 0.01 | 0.67 | 0.03 | 0.57 | 0.02 |
| FKH2 | 216 | 0.51 | 0.02 |  |  |  |  |
| GAT3 | 176 | 0.65 | 0.05 |  |  |  |  |
| GCN4 | 143 | 0.5 | 0.03 | 0.83 | 0.03 | 0.71 | 0.02 |
| GTS1 | 102 | 0.51 | 0.02 |  |  |  |  |
| HAP1 | 215 | 0.52 | 0.07 |  |  |  |  |
| HAP4 | 126 | 0.51 | 0.06 | 0.73 | 0.02 | 0.66 | 0.03 |
| HMS2 | 138 | 0.5 | 0.03 |  |  |  |  |
| INO2 | 114 | 0.5 | 0.01 |  |  |  |  |
| INO4 | 194 | 0.52 | 0.04 | 0.77 | 0.02 | 0.63 | 0.02 |
| MAC1 | 134 | 0.49 | 0.01 | 0.70 | 0.03 | 0.49 | 0.03 |
| MBP1 | 229 | 0.5 | 0.02 | 0.74 | 0.02 | 0.63 | 0.02 |
| MCM1 | 163 | 0.5 | 0.02 | 0.84 | 0.02 | 0.72 | 0.03 |
| MET32 | 154 | 0.5 | 0.02 |  |  |  |  |
| MGA1 | 188 | 0.49 | 0.01 |  |  |  |  |
| MSN4 | 145 | 0.5 | 0.02 | 0.63 | 0.04 | 0.49 | 0.03 |
| NDD1 | 190 | 0.54 | 0.01 |  |  |  |  |
| NRG1 | 136 | 0.49 | 0.03 | 0.73 | 0.03 | 0.57 | 0.03 |
| OAF1 | 169 | 0.5 | 0.02 | 0.62 | 0.04 | 0.50 | 0.02 |
| PDR1 | 163 | 0.61 | 0.03 |  |  |  |  |
| PHD1 | 166 | 0.5 | 0.01 |  |  |  |  |
| PHO2 | 141 | 0.53 | 0.05 |  |  |  |  |
| PHO4 | 165 | 0.49 | 0.02 | 0.53 | 0.04 | 0.49 | 0.02 |
| PIP2 | 111 | 0.5 | 0.01 |  |  |  |  |
| PPR1 | 106 | 0.51 | 0.03 |  |  |  |  |
| RAP1 | 408 | 0.65 | 0.02 | 0.89 | 0.01 | 0.83 | 0.02 |
| REB1 | 278 | 0.49 | 0.03 | 0.90 | 0.01 | 0.89 | 0.01 |
| RFX1 | 105 | 0.51 | 0.02 | 0.62 | 0.10 | 0.54 | 0.03 |
| RGM1 | 107 | 0.64 | 0.03 |  |  |  |  |
| RLM1 | 120 | 0.51 | 0.02 |  |  |  |  |
| RME1 | 107 | 0.54 | 0.03 |  |  |  |  |
| ROX1 | 172 | 0.5 | 0.05 |  |  |  |  |
| RPN4 | 176 | 0.5 | 0.01 | 0.64 | 0.03 | 0.51 | 0.02 |
| SKN7 | 166 | 0.52 | 0.03 | 0.85 | 0.03 | 0.64 | 0.03 |
| SMP1 | 181 | 0.6 | 0.02 |  |  |  |  |
| STB2 | 142 | 0.5 | 0.02 |  |  |  |  |
| STB4 | 117 | 0.54 | 0.05 |  |  |  |  |
| STB5 | 105 | 0.5 | 0.02 |  |  |  |  |
| STE12 | 130 | 0.5 | 0.01 | 0.79 | 0.03 | 0.67 | 0.02 |
| SUM1 | 121 | 0.5 | 0.04 | 0.87 | 0.03 | 0.75 | 0.03 |
| SUT1 | 172 | 0.5 | 0.01 |  |  |  |  |
| SWI4 | 252 | 0.56 | 0.05 | 0.85 | 0.02 | 0.68 | 0.02 |
| SWI5 | 201 | 0.52 | 0.03 |  |  |  |  |
| SWI6 | 230 | 0.52 | 0.03 |  |  |  |  |
| TEC1 | 114 | 0.51 | 0.03 |  |  |  |  |
| TYE7 | 105 | 0.49 | 0.04 |  |  |  |  |
| UME6 | 298 | 0.49 | 0.01 | 0.85 | 0.02 | 0.76 | 0.02 |
| UPC2 | 120 | 0.5 | 0.02 |  |  |  |  |
| WAR1 | 115 | 0.5 | 0.02 |  |  |  |  |
| YAP1 | 156 | 0.52 | 0.03 | 0.73 | 0.04 | 0.64 | 0.03 |
| YAP5 | 167 | 0.64 | 0.02 |  |  |  |  |
| YAP6 | 167 | 0.51 | 0.02 |  |  |  |  |
| YDR049W | 157 | 0.5 | 0.02 |  |  |  |  |
| YER130C | 178 | 0.51 | 0.02 |  |  |  |  |
| YFL052w | 112 | 0.5 | 0.03 |  |  |  |  |
| YLR278C | 112 | 0.51 | 0.03 |  |  |  |  |
| YOX1 | 111 | 0.51 | 0.01 |  |  |  |  |
| YRR1 | 157 | 0.51 | 0.02 |  |  |  |  |
