# Supplementary material for: Genome-wide analysis of chromatin features identifies histone modification sensitive and insensitive yeast transcription factors
Source: Genome Biol. 2011 Nov 7;12(11):R111. doi: 10.1186/gb-2011-12-11-r111 (PMC3334597; doi:10.1186/gb-2011-12-11-r111)
Supplement: Additional file 6 — Table S6. [file gb-2011-12-11-r111-S6.PDF]

Table S6. Histone sensitive and insensitive TFs

Histone sensitive TFs	Histone insensitive TFs
ABF1, ACE2, ARG80, ARG81, ASH1, AZF1, CAD1, CBF1, CIN5, CRZ1, CUP9, ECM22, FHL1, FKH1, FKH2, GAT1, GAT3, GCN4, GCR1, GCR2, GTS1, HAP1, HAP2, HAP4, HIR1, HIR2, HIR3, HMS1, INO2, INO4, LEU3, MAC1, MBP1, MCM1, MET31, MET4, MSN1, MSN4, NDD1, OPI1, PDR1, PHO2, PUT3, RAP1, REB1, RGM1, RLM1, RME1, ROX1, RPH1, SFP1, SKN7, SMP1, SPT2, STB1, STE12, STP1, SWI4, SWI5, SWI6, TBS1, TEC1, TYE7, YAP1, YAP5, YAP6, YML081W, ZAP1	A1, ABT1, ACA1, ADR1, AFT2, ARO80, ARR1, ASK10, BAS1, BYE1, CHA4, CST6, DAL80, DAL81, DAL82, DAT1, DIG1, DOT6, EDS1, FAP7, FZF1, GAL3, GAL4, GAL80, GLN3, GZF3, HAA1, HAC1, HAL9, HAP3, HAP5, HMS2, HOG1, HSF1, IFH1, IME1, IME4, IXR1, KRE33, KSS1, MAL13, MAL33, MBF1, MDS3, MET18, MET28, MET32, MGA1, MIG1, MIG2, MIG3, MOT3, MSN2, MSS11, MTH1, NDT80, NNF2, NRG1, OAF1, PDC2, PDR3, PHD1, PHO4, PIP2, PPR1, RCO1, RCS1, RDR1, RDS1, RFX1, RGT1, RIM101, RLR1, RPI1, RPN4, RTG1, RTG3, RTS2, SFL1, SIG1, SIP3, SIP4, SKO1, SMK1, SNF1, SNT2, SOK2, SPT10, SPT23, SRD1, STB2, STB4, STB5, STB6, STP2, STP4, SUM1, SUT1, SUT2, THI2, TOS8, UGA3, UME6, UPC2, USV1, WAR1, WTM1, WTM2, XBP1, YAP3, YAP7, YBL054W, YBR239C, YBR267W, YDR026C, YDR049W, YDR266C, YDR520C, YER051W, YER130C, YER184C, YFL044C, YFL052W, YGR067C, YHP1, YJL206C, YKL222C, YLR278C, YNR063W, YOX1, YPR022C, YPR196W, YRR1, ZMS1
