# Supplementary material for: Genome-wide analysis of chromatin features identifies histone modification sensitive and insensitive yeast transcription factors
Source: Genome Biol. 2011 Nov 7;12(11):R111. doi: 10.1186/gb-2011-12-11-r111 (PMC3334597; doi:10.1186/gb-2011-12-11-r111)
Supplement: Additional file 7 — Figure S1. [file gb-2011-12-11-r111-S7.PDF]

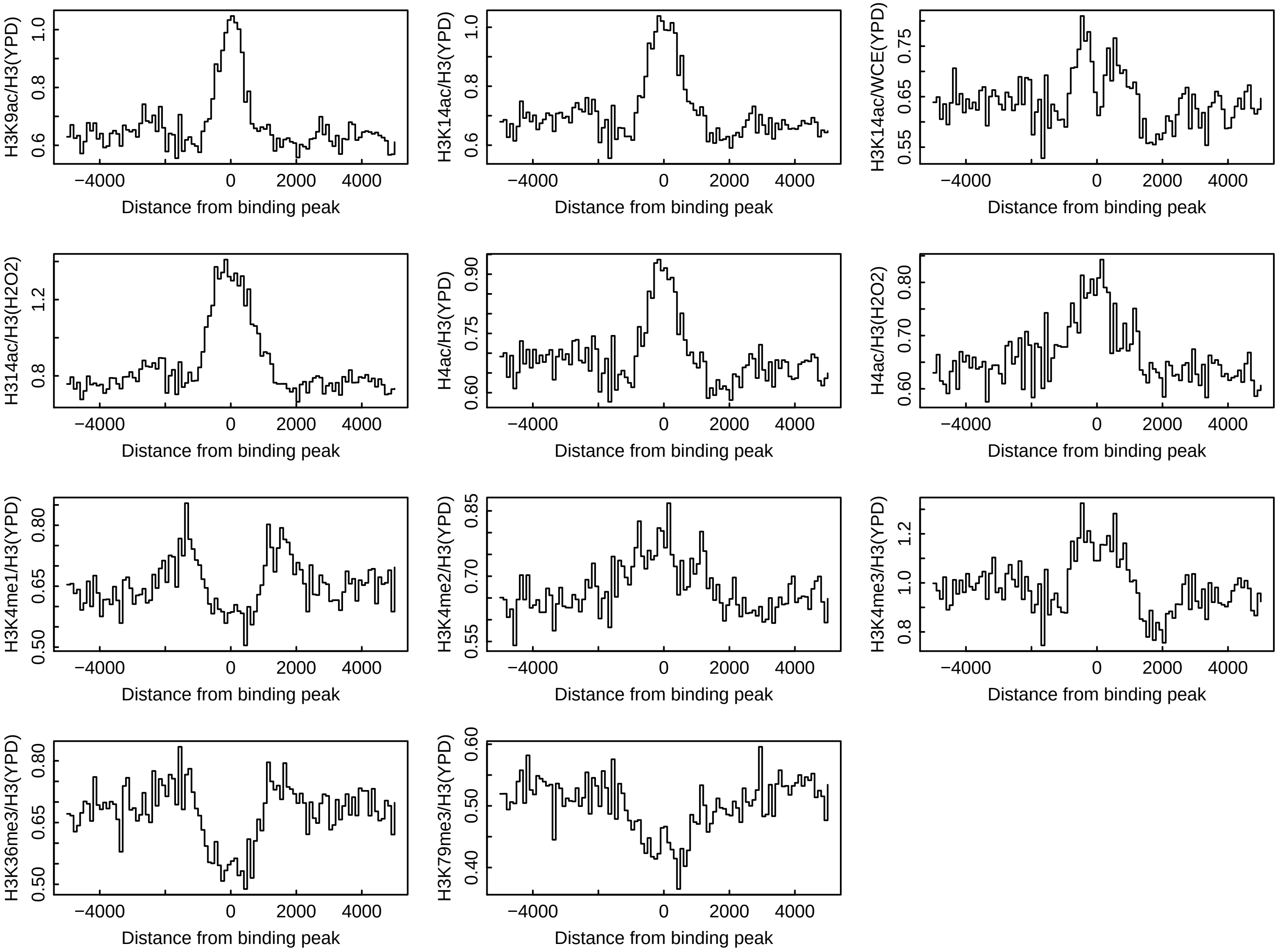
H3K9ac/H3(YPD)
0.6
0.8
1.0
−4000
0
2000
4000
Distance from binding peak
H3K14ac/H3(YPD)
0.6
0.8
1.0
−4000
0
2000
4000
Distance from binding peak
H3K14ac/WCE(YPD)
0.55
0.65
0.75
−4000
0
2000
4000
Distance from binding peak
H314ac/H3(H2O2)
0.8
1.2
−4000
0
2000
4000
Distance from binding peak
H4ac/H3(YPD)
0.60
0.75
0.90
−4000
0
2000
4000
Distance from binding peak
H4ac/H3(H2O2)
0.60
0.70
0.80
−4000
0
2000
4000
Distance from binding peak
H3K4me1/H3(YPD)
0.50
0.65
0.80
−4000
0
2000
4000
Distance from binding peak
H3K4me2/H3(YPD)
0.55
0.70
0.85
−4000
0
2000
4000
Distance from binding peak
H3K4me3/H3(YPD)
0.8
1.0
1.2
−4000
0
2000
4000
Distance from binding peak
H3K36me3/H3(YPD)
0.50
0.65
0.80
−4000
0
2000
4000
Distance from binding peak
H3K79me3/H3(YPD)
0.40
0.50
0.60
−4000
0
2000
4000
Distance from binding peak
